# Psychometric evaluation of a short-form version of the Swedish “Attitudes to and Knowledge of Oral Health” questionnaire

**DOI:** 10.1186/s12877-022-03215-z

**Published:** 2022-06-22

**Authors:** Maria Snogren, Amir H. Pakpour, Irene Eriksson, Malin Stensson, Kristina Ek, Maria Browall

**Affiliations:** 1 School of Health Sciences, University of Skövde, Skövde, Sweden; 2 Research School of Health and Welfare, Jönköping University, Jönköping, Sweden; 3 School of Health and Welfare, Jönköping University, Jönköping, Sweden; 4 Department of Nursing, School of Health and Welfare, Jönköping Academy for Improvement of Health and Welfare, Jönköping University, Jönköping, Sweden; 5 Centre of Oral Health, School of Health and Welfare, Jönköping University, Jönköping, Sweden; 6 Department of Oncology, Institute of Clinical Sciences, Sahlgrenska Academy, University of Gothenburg, Gothenburg, Sweden

**Keywords:** Attitudes, Healthcare professional, Knowledge, Older adults, Oral health, Oral health care

## Abstract

**Background:**

Healthcare professionals’ attitudes to and knowledge of oral health are fundamental to providing good oral health care to older adults. One instrument that assesses healthcare professionals’ attitudes to and knowledge of oral health in a Swedish context is the “Attitudes to and Knowledge of Oral health” (AKO) questionnaire. Two of the three item-groups of the AKO have previously been validated in a Swedish context. However, it is crucial that all three item-groups are validated, and beneficial to design a shorter, easy-to-use questionnaire for healthcare professionals while maintaining adequate integrity of its reliability and validity. Therefore, the present study aims to develop a short-form version of AKO and to secure its psychometric properties.

**Methods:**

Psychometric evaluation with Classical Test Theory and Item Response Theory to validate and shorten AKO with 611 healthcare professionals from a population of 1159 working in a municipality in an urban area in western Sweden.

**Results:**

Of the original 16 items in the AKO, 13 were shown to warrant retention in the abbreviated/shortened form. These showed acceptable validity and reliability for assessing healthcare professionals’ attitudes to and knowledge of oral health.

**Conclusion:**

This validated short-form version of AKO shows acceptable validity and reliability after being reduced to 13 items, structured in a 3-part scale. The items are consistent with the total scale, indicating that the internal consistency is acceptable. Future studies should be performed to evaluate AKO in other groups of healthcare professionals, across cultures, languages, and so on, to investigate its use and strengthen its validity and reliability.

## Background

Oral health is an integral part of general health and quality of life and is a fundamental human right [1], but is often a neglected area of healthy ageing [2, 3]. Poor oral health is a common cause of impaired general health [4] and one of the most costly forms of ill health [5] and is not a part of normal ageing [6]. Nevertheless, poor oral health is common among care-dependent older adults [7] and is associated with mortality, high age, malnutrition, being inactive, and cognitive impairments [8]. The World Dental Federation (FDI) defines oral health as being “multi-faceted and includes the ability to speak, smile, smell, taste, touch, chew, swallow, and convey a range of emotions through facial expressions with confidence and without pain, discomfort, and disease of the craniofacial complex” [9]. Good oral care habits, and receiving support from healthcare professionals, if needed, to perform oral care, are essential for good oral health among older adults [10]. Existing evidence shows that healthcare professionals working with older care recipients might neglect oral health and oral health care for various reasons, such as: lack of time and knowledge; lack of routines/guidelines or unclear division of responsibilities [10, 11]; attitudes to performing, or feelings of unpleasantness in performing, oral care and perceptions that it is a violation of integrity [12, 13]; or its low priority [14]. A recently published systematic review [15] emphasizes that regular inspection/assessments of older adults’ oral health by healthcare professionals is necessary to prevent poor oral health.

The instrument most commonly used in Sweden by healthcare professionals to assess older adults’ oral health is the Revised Oral Assessment Guide (ROAG) [16]. ROAG is a standardized measurement instrument developed for healthcare professionals to detect, examine, and document illnesses or problems in the mouth. ROAG evaluates oral health by assessing the condition of the voice, lips, oral mucosa, tongue, gums, teeth, saliva, swallowing, and any prostheses/implants [16]. However, ROAG does not assess healthcare professionals’ attitudes to and knowledge of oral health, which one multidisciplinary Delphi study [17] suggests is essential for providing good oral healthcare. Few instruments assess both healthcare professionals’ attitudes to and knowledge of oral health [18–21], and even fewer have been validated and tested for reliability [18, 20, 21]. Preston, Punekar and Gosney’s [19] instrument assesses healthcare professionals’ knowledge of and attitudes towards oral care and of providing oral health care to older adults. The Dental Coping Beliefs Scale (DCBS) has been translated to Swedish [20], but this scale assesses healthcare professionals’ oral healthcare priorities. Paryag, Rafeek and Lewis’ [18] instrument assesses knowledge, attitudes, and beliefs among healthcare professionals working in nursing homes. Other instruments are focused on parents’ [22] or dentists’ [23, 24] attitudes to and knowledge of oral health. One questionnaire that assesses healthcare professionals’ attitudes to and knowledge of oral health is the questionnaire, “Attitudes to and Knowledge of Oral health” (AKO), which was developed in a Swedish context by Paulsson, Fridlund, Holmén and Nederfors [21]. AKO has been used in a number of studies [10, 21, 25–28], but has only been validated once [21] with a sample of 60 nursing students. In that study, the reliability of AKO, estimated as internal consistency, was tested with Cronbach’s alpha. This showed a value of between 0.52 and 0.93, based on the different groups of questions and three factors, with an eigenvalue above 1.0 (with 59.1 total variance). AKO contains sixteen items organized into three groups. Paulsson, Fridlund, Holmén and Nederfors [21] validated only two of the groups, “Knowledge of importance” and “Implementation possibilities”, but not “Attitudes to oral hygiene.” Paulsson, Fridlund, Holmén and Nederfors [21] describe the items in “Attitudes to oral hygiene” as background items. However, we argue that it is important to validate each of the AKO’s three item groups with healthcare professionals as, according to Paulsson, Fridlund, Holmén and Nederfors [21], this is AKO’s target group. There is also a need to design a meaningful yet shortened version of the AKO questionnaire without compromising the integrity of its reliability and validity, because validated, short, simple, and easy-to-use questionnaire usually attract higher response rates than do long, complex ones [29, 30]. With the aim of reducing respondent burden, there is also a need to design a meaningful yet shortened version of the AKO questionnaire. Therefore, the present study aims to develop a short-form version of AKO and to secure its psychometric properties.

## Methods

### Design

A psychometric evaluation with Classical Test Theory (CTT) and Item Response Theory (IRT) was used to validate the self-administered AKO questionnaire and design a shortened version [31, 32].

### Data collection and ethics

AKO was administered to healthcare professionals working in a municipality in an urban area in western Sweden in the spring of 2013. The municipality has the overall responsibility for providing nursing care in both home healthcare services and municipality-run nursing homes. Participants were recruited by giving written and verbal information about the study to the head of healthcare services for the community, who identified and asked the informants to participate. The inclusion criterion was healthcare professionals (nurses or nursing assistants) who worked 50% or more. Healthcare professionals (*n* = 1159) from five different settings were approached and asked to participate. The address of each participant was obtained from the healthcare service head. The participants were then posted a letter with a copy of the AKO along with information about the study and a pre-paid return envelope. After the initial distribution of questionnaires (*n* = 1159), a reminder was sent to 595 participants who had not replied after four weeks. The total number of returned questionnaires were 616, and 611 (53%) had valid responses and were included in the analysis. The study was approved by the Swedish Ethical Review Authority in Gothenburg (Dnr: 891:13).

### Participants

The participants (*n* = 611) included in the present study were mostly women (95%). At the time of data collection, they were aged from 26–66 years with a mean age of 49 years. The sample comprised mostly assistance nurses (94%) and nurses (6%), with several years of professional experience, ranging from 4–40 years with an average of 18 years. More than half (60%) worked 75% or more, and 62% worked both day- and night-time, while 29% worked only during the daytime, and 6% only at night-time. Most participants worked in nursing homes (76%), 12% in home care, and 8% in short-term care.

### Attitudes to and knowledge of oral health questionnaire

AKO were developed by Paulsson, Fridlund, Holmén and Nederfors [21], all of whom are experienced in older adults’ care. AKO contains sixteen statements about attitudes to and knowledge of oral health within three subject groups: 1. *Attitudes to oral hygiene*, four items graded on a Likert scale from 1 (never) to 5 (always); 2. *Implementation possibilities*, six items on a Likert scale from 1 (never) to 5 (always); and 3. *Knowledge of importance*, six items on a Likert scale from 1 (unimportant) to 5 (important) (Table 1). AKO also contains background data such as gender, age, occupation, years in the profession, and level of education (nominal scale).

**Table 1:** Items in the AKO

**Attitudes to oral hygiene**
** Item 1-** I think it feels nasty to take care of other people’s mouths
Item 2- I think oral care is part of my job duties
** Item 3**- I think it is practically difficult to perform oral care
** Item 4**- The caregiver refuses to receive help with oral care
**Implementation possibilities**
What opportunities do you think you have when it comes to offering oral care to the healthcare provider you are responsible for?
** Item 5**- I can take the time needed to provide oral care
** Item 6**- I have enough knowledge to perform proper oral care
** Item 7**- I have appropriate aids for the implementation of proper oral care
Item 8- I know how to practically perform oral care
** Item 9**- To caregivers who want to take care of their oral care themselves, I can give appropriate oral care advice
Item 10- By actively informing “reluctant” caregivers, I can in the long run get them to accept help with oral care
**Knowledge of importance**
What skills do you think are important for being able to perform good oral care?
** Item 11**- Assistive products and oral care
** Item 12**- Diseases affecting the oral cavity
** Item 13**- Various artificial (prosthetic) dental substitutes
** Item 14**- What the healthy oral cavity looks like
** Item 15**- Oral physiological function (e.g., chewing, swallowing, speech)
** Item 16**- The psychosocial function of the oral cavity (e.g., appearance, well-being)

AKO (Attitudes to and Knowledge of Oral health questionnaire)

Highlighted items are the 13 items that remain and should be included in the short version of the questionnaire

### Data analysis

There are two main approaches to evaluating psychometric properties: Classical Test Theory (CTT), and Item Response Theory (IRT). CTT relates to Spearman’s concept of the observed score, including true and false components. This theory’s foundation is based on an equation in which the observed score (obtained) is hypothetically composed of a true score and an error score. In this model, the true score for each person is constant and will not change in repeated measurements [31, 32].

The limitations of CTT include the following:

- 1. In this theory, the indicators related to the test and the questions depend on the sample group. The sample group’s level of ability and its distribution strongly affects the item characteristics, such as diagnostic power, difficulty level, standard deviation, variance, and test mean. For this reason, the ability to generalize the results to other groups and communities is limited.
- 2. Revealing the level of ability of people depends on tests and questions. A person may score differently on two tests that measure the same trait but differ in difficulty levels.
- 3. The test, which is based on classical theories, is aimed more at people with moderate levels of ability. This means that the validity of the test is lower in both the upper-ability group and in the lower-ability group than in the middle group.
- 4.The standard measurement error is assumed to be the same for all individuals. This assumption leads to wrong decisions about people because standard error can vary based on ability level.
- 5. The degree of difficulty cannot be exactly the probability of answering a certain question correctly among people with different abilities.

Overcoming these shortcomings and limitations of CTT has led to the development of IRT.

IRT-derived models are used to develop tests, align non-parallel test scores, examine question bias, and report scores. IRT is based on a fundamental one-dimensional attribute that is measured by testing [33].

The Rasch model (as an IRT model) includes a model-based assessment, in which the assessment – the level of ability – depends on the responses of the individuals and the characteristics of the items in which the test was developed [34].

In this study, we used both CTT and IRT approaches to assess the psychometric properties of AKO. As for the CTT, floor and ceiling effects, confirmatory factor analysis (CFA), internal consistency, and corrected item-total correlation were computed. Floor and ceiling effects were measured by calculating the percentage of participants who reported the highest (ceiling) and the lowest (floor) possible score for each subscale, with < 20% as the acceptable threshold. Cronbach’s alpha was computed to assess the internal consistency of the AKO subscale, with values of 0.70 or higher as the acceptable threshold [35].

CFA was conducted by using a diagonally weighted least squares (DWLS) estimation to test the proposed three-facture structure of AKO. Several model fit indices were used to examine the proposed factor structure of AKO: Comparative fit index (CFI), Tucker-Lewis index (TLI), and root mean square error of approximation (RMSEA). The recommended cut-off criteria for the CFI, TLI and RMSEA were > 0.9, > 0.9, and < 0.08, respectively [36].

Factor loadings extracted from the CFA were used to compute average variance extracted (AVE) and composite reliability (CR). Values higher than 0.5 and 0.6 were considered satisfactory for AVE and CR, respectively.

Regarding to the IRT, Rasch analysis with a partial credit model was performed on the data. The items of AKO were analyzed using information-weighted fit statistic (infit), mean square (MnSq), and outlier-sensitive fit statistic (outfit) MnSq, with values of between 0.5 and 1.5 deemed acceptable. Moreover, the reliability of the AKO subscale was examined using item and person separation reliability (acceptable value > 0.7); item and person separation index (acceptable value > 2) [37].

Identifying problematic items through item analysis plays an important role in a test. Therefore, differential item functioning (DIF) was computed for each item of AKO to assess whether the items varied across gender groups. Values higher than 0.50 were considered to be significant DIF [38]. All analyses were carried using SPSS version 25.0, version 3.5.20 and WINSTEPS, version 4.3.0.

## Results

Cronbach’s alpha for the full AKO, with 16 items grouped into Attitudes to oral hygiene, Implementation possibilities, and Knowledge of importance, were, 0.275, 0.806, and 0.869, respectively. After item 2 was omitted from the attitude group, internal consistency for that group improved, as Cronbach’s alpha was increased to 0.460.

The results of the CFA are illustrated in Table 2. The hypothesized three-factor model of AKO with 15 items fit with the data (CFI = 0.985, TLI = 0.982, and RMSEA = 0.056). However, factor loading values for items number 8 and 10 were low (i.e., 0.251 and 210, respectively). Therefore, these two further items were omitted and the modified three-factor model (after removing items number 2, 8, and 10) of AKO had satisfactory fit indices (CFI = 0.998, TLI = 0.997, and RMSEA = 0.011). The standardized factor loading values for the final modified model were significant and ranged between 0.317 for item 4 and 0.794 for item 14. Moreover, intercorrelation between the latent factors ranged from 0.126 to 0.526. Cronbach’s alpha coefficient ranged from 0.460 (Attitudes to oral hygiene) to 0.869 (Knowledge of importance) between domains, and the corrected item-total correlation ranged from 0.214 for item 4 to 0.732 for item 16.

**Table 2:** Psychometric properties of the remaining items in the revised AKO

Item	Analyses from classical test theory		Analyses from Rasch				
	Factor loading^a^	Item-total correlation	Infit MnSq	Outfit MnSq	Discrimination	Difficulty	DIF contrast across gender^bc^
**Item 1**	0.543	0.226	1.24	1.14	0.83	-1.68	0.16
**Item 3**	0.512	0.396	0.77	0.78	1.19	0.84	-0.06
**Item 4**	0.317	0.214	0.96	1.00	0.97	0.84	-0.09
**Item 5**	0.551	0.448	1.43	1.42	0.54	-0.41	0.08
**Item 6**	0.793	0.668	0.70	0.70	1.35	-0.10	-0.16
**Item 7**	0.756	0.618	0.79	0.80	1.23	0.350	0.37
**Item 9**	0.617	0.502	1.08	1.08	0.90	0.01	-0.27
**Item 11**	0.601	0.556	1.22	1.37	0.73	-0.52	0.05
**Item 12**	0.703	0.665	0.96	0.95	1.07	-0.27	-0.07
**Item 13**	0.697	0.640	1.21	1.24	0.77	0.60	0.43
**Item 14**	0.794	0.726	0.84	0.83	1.18	-0.29	-0.05
**Item 15**	0.757	0.705	0.93	0.94	1.09	0.20	-0.15
**Item 16**	0.791	0.732	0.80	0.80	1.20	0.27	-0.35

AKO (Attitudes to and Knowledge of Oral health questionnaire)

^a^ Based on confirmatory factor analysis

^b^ DIF contrast > 0.5 indicates substantial DIF

^c^ DIF contrast across gender = Difficulty for males-Difficulty for females

*MnSq*  Mean square error, *DIF* Differential item functioning

AVE and CR values for the shortened AKO subscales, Implementation possibilities and Knowledge of Importance, were acceptable, but the subscale for Attitudes to oral hygiene did not have acceptable AVE and CR values (0.22 and 0.44, respectively) (Table 3).

**Table 3:** Psychometric properties of the remaining items in the revised AKO (after omitting item numbers 2, 8, and 10) at scale level

	AT^a^	IP^b^	KI^c^	Suggested cutoff
**Psychometric testing**				
Ceiling effects (%)	1.3%	2.0%	39.9%	< 20
Floor effects (%)	0.2%	0.2%	0	< 20
Internal consistency (Cronbach’s α)	0.460	0.760	0.869	> 0.7
**Confirmatory factor analysis**				
χ^2^ (*df*)	66.372 (62)	-	-	Nonsignificant
Comparative fit index	0.998	-	-	> 0.9
Tucker-Lewis index	0.997	-	-	> 0.9
Root-mean square error of Approximation	0.011 (0.00–0.028)	-	-	< 0.08
Standardized Root Mean Squared **Residual (SRMR)**	0.042	-	-	< 0.08
Average Variance Extracted	0.22	0.47	0.65	> 0.5
Composite Reliability	0.44	0.78	0.92	> 0.6
Item separation reliability from Rasch	1.00	0.96	0.95	> 0.7
Item separation index from Rasch	18.17	4.69	4.37	> 2
Person separation reliability from **Rasch**	0.70	0.71	0.54	> 0.7
Person separation index from Rasch	2.00	2.03	1.08	> 2

AKO (Attitudes to and Knowledge of Oral health questionnaire)

^*^p < 0.001

^a^ Attitudes to oral hygiene (Items 1, 3, 4)

^b^ Implementation Possibilities (Items 5, 6, 7, 9)

^c^ Knowledge of Importance (Items 11–16)

The most ambiguous items were items 3 and 4 because of their logit values were + 0.84, and the most significant item was item 1 because of its logit value of -1.68.

The results of the Rasch analysis are reported in (Table 2). All infit and outfit MnSq values were within the acceptable range of 0.70 to 1.43.

Based on the results of Rasch analysis (Table 2), three items from the original 16 included in AKO also warranted exclusion: item 2, item 8, and item 10. Items 2, 8, and 10 were excluded because their infit and outfit values were outside of the recommended range (i.e., 1.61, 1.56, and 1.58 for item 2, item 8, and item 10, respectively). The recommended items to include are highlighted in Table 1 (items 1, 3, 4, 5, 6, 7, 9, 11, 12, 13, 14, 15 and 16). The results of the Rasch analysis for the shortened AKO (with 13 items) are reported in Table 2. All infit and outfit MnSq values were within the acceptable range of 0.70 to 1.43.

Items in the shortened AKO were invariant across different groups in relation to gender. Therefore, both male and female participants had similar perceptions towards all AKO items.

## Discussion

The current study aimed to secure psychometric properties and develop a shortened form of AKO. The study showed that removing three items from the original version of the AKO produced an instrument with acceptable validity and reliability.

The intention was to produce a robust and succinct but delimited instrument to assess healthcare professionals’ attitudes to and knowledge of oral health. The main reason for designing a shortened form of AKO was to increase its usability and usefulness for testing health care professionals’ attitudes and knowledge of oral health in a Swedish context.

As described in the background section, the target group for AKO is healthcare professionals. Therefore, we chose a large sample (*n* = 611) of healthcare professionals, in contrast to the sample used in the earlier validation performed by Paulsson, Fridlund, Holmén and Nederfors [21], as it is essential to carry out validation with the right target group and an appropriate number of participants to obtain accurate calculations [39]. A systematic review from 2018 [32] also describes that it is not enough to determine a questionnaire’s robustness solely by measuring Cronbach’s alpha, which was used to test internal consistency, alongside an exploratory factor analysis to test construct validity, in AKO’s earlier validation [21]. Therefore, we adopted two approaches to perform the psychometric testing of the AKO: CTT and IRT. In CTT, items can be summed (without weighting or standardization) to obtain a total score (e.g., mean values and SDs). In IRT, the locations of persons and items on a latent continuum are measured [40]. CTT and IRT thus complement each other by facilitating different calculations of AKO. However, the present study showed that, by removing three problematic and ambiguous items (2, 8, and 10), the shortened version of AKO showed acceptable validity and reliability according to the CTT and IRT. Item 2 can be seen as an advantageous background question, with answer options yes or no, instead of an item in the questionnaire, because of its importance in determining healthcare professionals’ attitudes to whether oral care is part of their job duties. Items 8 and 10 are seen to be included in other items (item 8 in 3, 6 and 7; and item 10 in 5, 7, and 9) in the questionnaire and can therefore be excluded. The AVE and CR values for the group Attitudes to Oral health (items 1, 2 and 4) showed unacceptable values in the current study. Despite this, the subscale is interpreted as being essential to assess healthcare professionals’ attitudes to oral health and, therefore, should be included in AKO.

AKO assesses healthcare professionals’ attitudes to and knowledge of oral health and provides a picture of these to determine whether knowledge gaps exist and whether training/education efforts concerning oral health care are necessary for healthcare professionals. ROAG [15], which measures older adults’ oral health, and AKO can complement each other and provide healthcare professionals with important knowledge. This is because understanding healthcare professionals’ attitudes to and knowledge of oral health is essential in providing good oral health care, promoting good general health and quality of life for older adults, and in helping them avoiding pain and infection, and reducing suffering and oral health-associated mortality [10, 41]. The AKO short-form version proposed here can help healthcare professionals to realize the vital role that oral health plays in promoting good quality of life and general health among older adults and in reducing poor oral health. This short-form version of AKO has been validated, is easily accessible, and is easy to complete. It includes 13 items and can be used in larger studies to assess healthcare professionals’ attitudes to and knowledge of oral health in a Swedish context. As previously described, validated, short, simple, and easy-to-use questionnaires usually attract higher response rates than do long, complex ones [29, 30]. Therefore, this AKO short-form version can be an important way to describe healthcare professionals’ attitudes to and knowledge of oral health.

This study’s main strength is the large sample size of healthcare professionals, who are, as Paulsson, Fridlund, Holmén and Nederfors [21] describe, the target group for AKO. The use of both CTT and IRT approaches to test the psychometrics qualities of AKO is a further strength. However, some limitations must be considered when interpreting the results. One is that there may be problems when generalizing the results to other health care professionals, as the context for this study was professionals working with older adults. A second limitation can be seen in the lack of a test–retest component. The current study did not perform an analysis of the factors that affected the low response rate because of the lack of information about the informants who chose to not participate. As such, we were unable to determine how our sample was distributed or whether it was representative of the wider population, which can be seen as a third limitation. A fourth limitation might be the age of the data. However, the data have not been published before and no other re-validations have taken place since the data were collected, and, as our literature review revealed [10, 13, 17, 42], healthcare professionals’ attitudes to and knowledge of oral health are essential for providing oral health care. Therefore, in future studies AKO should be evaluated in other groups of healthcare professionals aiming to investigate AKO in broader use, using a test–retest method in the design and collecting data with healthcare professionals to improve the use of AKO.

## Conclusions

This short-form version of AKO shows acceptable validity and reliability to assess healthcare professionals’ attitudes to and knowledge of oral health. Our findings suggest that the AKO is best structured as a three-part scale comprising 13 items. The items are consistent with the total scale, indicating that the internal consistency is acceptable if three items (2, 8 and 10) are excluded from the original AKO version. Future studies should be performed to evaluate the questionnaire in other groups of healthcare professionals, across different cultures, in different languages, and so on, to further investigate the use of AKO and to strengthen its validity and reliability.

## Data Availability

The data supporting this study's findings are available by application to the corresponding author. Still, restrictions apply to the availability of these data, which were used under license for the current study, and so are not publicly available. Data are, however, available from the authors upon reasonable request.
